# A state-space model to derive motorboat noise effects on fish movement from acoustic tracking data

**DOI:** 10.1038/s41598-021-84261-2

**Published:** 2021-02-26

**Authors:** Margarida Barcelo-Serra, Sebastià Cabanellas, Miquel Palmer, Marta Bolgan, Josep Alós

**Affiliations:** 1 Institut Mediterrani d’Estudis Avançats, IMEDEA (CSIC–UIB), C/Miquel Marquès, 21, 07190 Esporles, Illes Balears Spain; 2 Laboratory of Functional and Evolutionary Morphology (Freshwater and Oceanic Sciences Unit of Research), Institut de Chimie, B6c, University of Liege, 4000 Liege, Belgium

**Keywords:** Behavioural ecology, Conservation biology, Marine biology

## Abstract

Motorboat noise is recognized as a major source of marine pollution, however little is known about its ecological consequences on coastal systems. We developed a State Space Model (SSM) that incorporates an explicit dependency on motorboat noise to derive its effects on the movement of resident fish that transition between two behavioural states (swimming vs. hidden). To explore the performance of our model, we carried out an experiment where free-living *Serranus scriba* were tracked with acoustic tags, while motorboat noise was simultaneously recorded. We fitted the generated tracking and noise data into our SSM and explored if the noise generated by motorboats passing at close range affected the movement pattern and the probability of transition between the two states using a Bayesian approach. Our results suggest high among individual variability in movement patterns and transition between states, as well as in fish response to the presence of passing motorboats. These findings suggest that the effects of motorboat noise on fish movement are complex and require the precise monitoring of large numbers of individuals. Our SSM provides a methodology to address such complexity and can be used for future investigations to study the effects of noise pollution on marine fish.

## Introduction

The oceans are filled with sounds generated by a variety of natural sources, including biotic sources such as mammals, fish and invertebrates^1–3^, and abiotic sources such as breaking waves, earthquakes, wind and rain^4^. However, many forms of underwater sounds are generated by anthropogenic sources, such as vessels, motorboats, wind turbines, military sonars, oil or gas drilling and oceanographic research technologies^5,6^. The negative consequences of high-intensity sounds generated by pile driving and seismic air guns on behaviour have been described mainly on marine mammals^7–9^. Over the years, however, research has broadened the scope and there has been an increase in the number of studies analysing the impacts of noise pollution from different sources on the ecology of marine fauna at different levels^10–12^.

The noise pollution emitted by recreational motorboats in coastal systems has generated recent interest^6,13^. The potential effects of noise emitted by motorboats on marine fish behaviour have been recently reviewed in^6^. Evidence suggests that some marine fish, when disturbed by motorboat noise stop moving and hide into shelters^14,15^, modify parental care related behaviours^16^, modify calling behaviour^17^, change female mate choice preferences^18^ and alter interspecific cooperation^19^. Furthermore, noise pollution may interfere with the animal’s ability to detect naturally generated sounds (i.e., masking), reducing the range to detect communicative sounds and other acoustic cues^19–22^. This can have a negative effect on communication for reproductive purposes, orientation and predator or prey detection, potentially affecting survival and reproduction^23,24^. All these findings highlight the importance of measuring the impact of motorboat noise on marine fauna for management and conservation of marine coastal areas^12^.

The effects of motorboat noise on fish movement and space use have received less attention. The lack of proper technology to measure fish movement in the wild has limited our ability to disentangle the consequences of noise pollution on fish movement behaviour. Movement and space use constitute behavioural traits that when measured in the wild provide major insight into the biology, ecology and conservation of marine fish species^25^. In fact, some of the above mentioned studies exploring the effects of anthropogenic noise on fish behaviour have been carried out under laboratory conditions^18,20,21,23^. Captive animals can show altered patterns of behaviour and the sound transmission inside confined areas might not have the same physical parameters than natural sounds in the wild^6^. Although laboratory experiments are fundamental and provide answers that cannot be reached in field studies (e.g., hearing threshold shifts and auditory and hormonal effects), there are limitations when extrapolating the laboratory results and applying the conclusions to wild populations. In order to extract robust conclusions on the ecological consequences of motorboat noise on marine fish, there is a need to combine laboratory results with measures of movement and space use in wild fish exposed to real sources of noise pollution in their natural environment.

Acoustic telemetry technology (i.e., acoustic tracking) is based on applying, to the studied organism, micro acoustic transmitters emitting acoustic signals that are received by an array of acoustic receivers^25,26^. Acoustic tracking has been developed and widely used to describe the movement, territory size, home range, space use and habitat selection in many coastal fish species in wild settings^27^. However, there is substantial evidence that the acoustic tracking of an individual can be highly influenced by several factors such as water currents, tidal phase, environmental noise, distribution of the array of receivers and distance between the emitter and receiver units^28,29^. In order to use this technology in behavioural studies, the development of specific statistical solutions is required to solve the issues associated with the large observational error^30^.

State-space models (SSM) have emerged as one of the most promising tools to study animal movement in the wild while properly addressing the limitations of tracking uncertainty^29,30^. Furthermore, they are a flexible tool that allows for the integration of environmental explanatory variables expected to affect animal movement^31^. Applying SSM to acoustic tracking data allows for the extraction of relevant behavioural information while considering the large observational error of the system^30^. SSM combine a behaviourally mediated process model (movement model), with an observation error model^29^. The movement model predicts the position of a tagged individual at a given time. This model can include different behavioural states (e.g., swimming or hidden), as well as any other environmental covariates that may affect the transition matrix among the behavioural states (e.g., noise pollution), making this model particularly attractive to test the effects of motorboat noise on fish movement. Finally, the error model predicts the probability of detecting a tagged individual by a receiver in function of the distance between them (see details in^30^).

Given this background, the objective of this work was two-fold. First, we developed an improved analytical tool to properly integrate the effects of environmental variables (presence of noise peaks generated by motorboats passing at close range and diel pattern of behaviour) on a two-state movement model (swimming vs. hidden) for marine resident fish. Second, we tested the hypothesis whether or not the noise peaks generated by motorboats passing at close range (within the study area) affected the transition probabilities between the two behavioural states in the species *Serranus scriba* (Linnaeus, 1758). *S. scriba* is a small-bodied Serranid, resident of littoral marine waters of the Mediterranean and Black sea that establishes a home range or bounded area to perform its vital activities^32,33^. *S. scriba* is a highly valued species in recreational fisheries at the NW Mediterranean subject to high fishing pressures in places such as the Balearic Islands^34^. For the purpose of this work, we monitored the movement of individuals via acoustic tracking and developed a SSM built upon the works of Alós et al.^30^ and Palmer et al.^35^ to analyse the data. We predicted that the noise peak generated by motorboats passing at close range can alter the movement behaviour of coastal fish species, with fish seeking refuge under such circumstances (hide or fear effect)^36^, having an impact on macroscopic patterns of space use. This work is not only important for providing a methodological solution to study the effects of motorboat pollution on animal behaviour, but could be used as a tool for making informed managerial decisions in coastal areas, and more specifically Marine Protected Areas.

## Methods

### Development of the space state model

**Figure 1:**
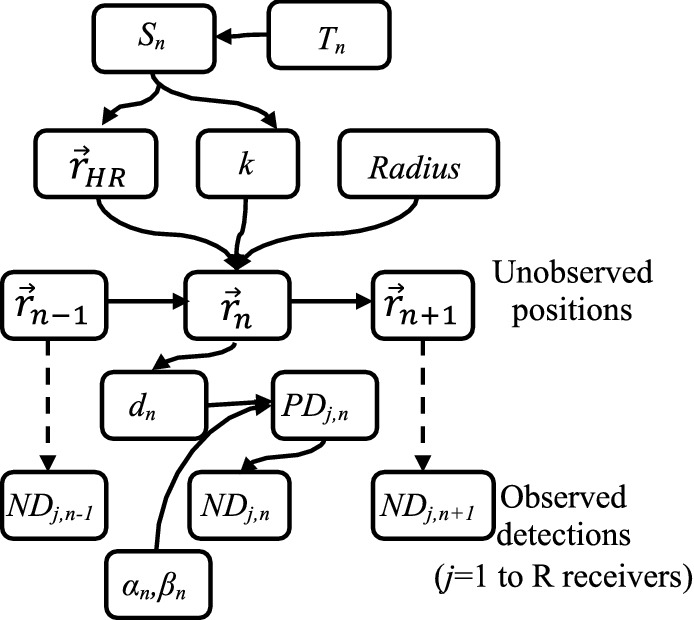
Directed acyclic graph of the State-Space Model (SSM). The unobserved position ${\overrightarrow{r}}_{n}$ at time step *n* is generated following a combination of movement parameters (the process model) of the fish: position of the centre of the home range (${\overrightarrow{r}}_{HR}$), strength of the driving force attracting the individual to the centre of the home range (*k)* and *Radius* of the home range. The actual position (*n*) depends on the previous position (*n-1*), and the behavioural state (Sn; swimming vs. hidden) at time step *n* and is determined by the transition matrix *T*_*n*_ (Eq. 1, see main text). The observed data (number of detections, ND) at time *n* consists of all the detections recorded by each one of the receivers (*j* in R). Note that ND at time* n* is independent of the ND at time *n-1* and is generated using the probability of detection by receiver *j* at *n* time unit (*PD*_*j,n*_) determined by a logit function (with parameters *α* and *β* at time *n*) of the distance (*d* at *n*) between the (unobserved) fish position and the (known) receiver position (observational model).

For the theoretical component of this study, we developed a State Space Model (SSM) to model the movement of resident fish that transition between two behavioural states (swimming vs. hidden). The SSM incorporates an explicit dependency on the noise produced by motorboats to derive its effects on fish movement (Fig. 1). The alternate state movement pattern (swimming vs. hidden) has been widely described in resident marine fish transiting from swimming while searching for food to hiding to avoid predators^37^, and is particularly evident in our study case species *S. scriba*^38^.

**Figure 2:**
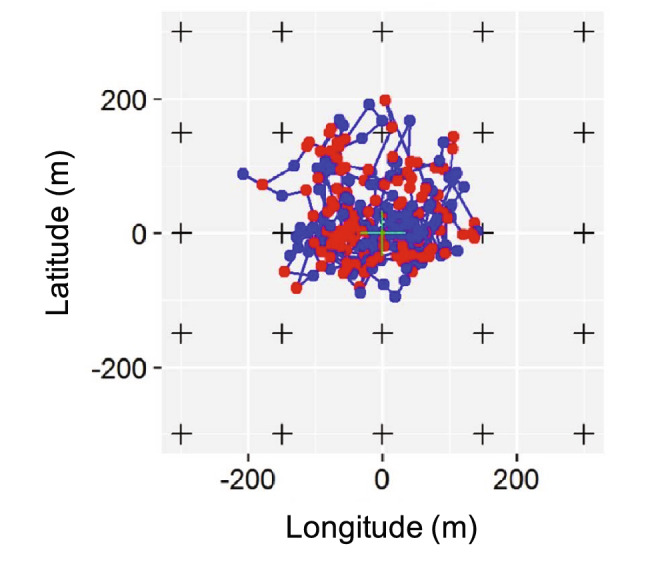
Simulation of 1000 time steps of a fish moving according to the two-state movement model tracked by an array of acoustic receivers. In red the positions of the fish while hidden and in blue the positions and trajectories when the fish is swimming. The black crosses represent the acoustic receivers. Note the aggregated distribution of positions imposed by the fish being limited to move within its home range area.

**Figure 3:**
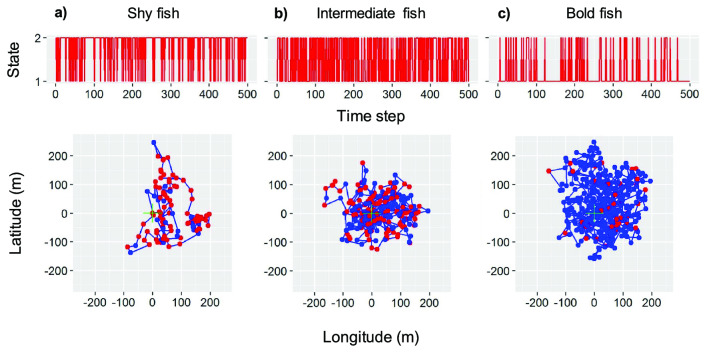
Simulation of 1000 time steps of three fish moving according to the two-state movement model with different probabilities of transition. Graphs on top show the Markovian-based transitions between state 1 (swimming) and state 2 (hidden) over time for each individual (shy, intermediate and bold). The scatter plots below represent the movement pattern. Red dots denote the location of a hidden individual while blue dots and lines represent individuals swimming and their path. The transition between states was generated using the transition matrix in Eq. (1). (**a**) graphs obtained from simulating data on shy fish (individuals that tend to remain in state 2, hidden); (**b**) simulated data on intermediate fish (individuals that do not show a preference for a state); and (**c**) simulated data on bold fish (individuals that tend to remain in state 1, swimming).

The model developed here is an adaptation for behavioural particularities of the SSM developed in^30^. The basic (i.e., with no covariates) movement model describing the expected movement pattern of coastal fish displaying home range behaviour assumes that fish follow a biased random walk (BRW)^39^. BRW considers that fish swim following random stimuli but with an additional tendency to remain close to a specific point defined as the centre of their home range^40,41^. According with this movement model (Fig. 1), fish spend most of the time within a bounded area because a drift force (*k*) pulls them towards the centre of the home range or shelter, limiting their exploratory range (Fig. 2). We described a function to determine the transition between the two states, swimming and hidden (Eq. 1). When swimming, fish move according to a BRW model while when hidden fish do not move (i.e., maintain the previous position). The transitions between the two behavioural states have been modelled with a transition matrix (Eq. 1):

1 $${T}_{n}=\left(\begin{array}{cc}{p}_{n}& 1-{p}_{n}\\ 1-{q}_{n}& {q}_{n}\end{array}\right)$$

where *p*_*n*_ is the probability to continue swimming at time *n* given that the fish was swimming at the previous time step (*n-1*); *1-p*_*n*_ is the probability of shifting from swimming to hidden at time *n* given that the fish was swimming at the previous time step (*n-1*); *q*_*n*_ is the probability to continue hidden at time *n* given that the fish was hidden at the previous time step (*n-1*); and *1-q*_*n*_ is the probability of shifting from hidden to swimming at time *n* given that the fish was hidden at the previous time step (*n-1*). The results of applying different transition matrices are demonstrated by simulated paths of fish moving with the same BRW but with different combinations of *p* and *q* (Fig. 3). The simulations show fish that tend to remain hidden (shy fish), fish with no preference towards a specific behaviour (intermediate fish) and fish that tend to swim (bold fish, Fig. 3).

In our study, two putative explanatory variables were considered to affect *p* and *q*: the natural diel pattern of fish behaviour and the presence of noise peaks generated by passing motorboats at close range. They were both considered categorical variables, day vs. night and presence vs. absence of motorboats passing at close range. The first variable was obtained using the sunrise and sunset hours in UTC, while the second one was extracted from sound recordings (see section below for details on the methodology). Accordingly, four different *T*_*n*_ matrices were defined: (1) day and noise (at daylight in presence of noise peaks generated by passing motorboats), (2) day and silence (at daylight in absence of noise peaks generated by passing motorboats), (3) night and noise (at night in presence of noise peaks generated by passing motorboats), and (4) night and silence (at night in absence of noise peaks generated by passing motorboats). For each one of the four matrices, the parameters *p*_*n*_ and *q*_*n*_ were estimated (for a total of 8 estimated parameters). For convenience (see details in the next section), in this work the noise generated by passing motorboats was transformed into a binary variable (presence vs. absence of noise peaks generated by passing motorboats). Note, however, that the model can be easily accommodated to fit any other continuous explanatory variable (e.g. noise levels in dB). In this case, the natural choice would be a linear combination at the logit scale, resulting in a sigmoidal response between 0 and 1. For example, to estimate *p* at daylight one could apply: logit(*p*_*n*_) = *β*_*1,p,day*_ + *β*_*2,p,day*_*V*_*n*_, where *V*_*n*_ is the (continuous) value of the putative explanatory variable at time *n.*

Our two-state movement model was designed to be fed by acoustic tracking data generated by an array of receivers, requiring for the integration of an observational error module (Fig. 1). The raw data obtained from acoustic tracking experiments are not fish positions, but a number of detections at a given time period by every one of the acoustic receivers (Fig. 2). Alós et al.^30^ described a logistic function to extract fish positions from such detections. Briefly, the number of detections for a given individual during a specific period is assumed to be binomially distributed, with a probability of detection depending on a logistic function of the distance between the individual and a receiver (Fig. 1). We estimated the parameters of the logistic function within our SSM framework model using the acoustic tracking data described below.

### Acoustic tracking and noise data collection

**Figure 4:**
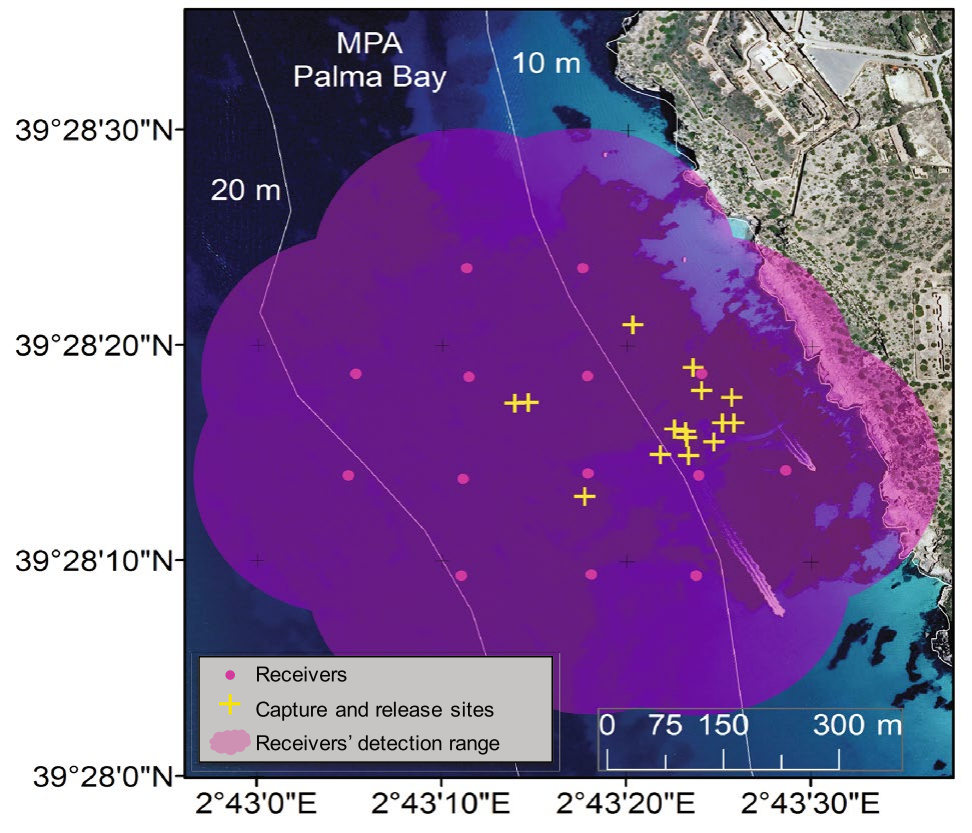
Map of the study area showing the acoustic tracking experimental setup. The map shows the distribution of the acoustic receivers (pink dots) deployed within the no-take Marine Protected Area (MPA) of Palma Bay (Mallorca, Spain), as well as the expected detection range of the receivers (shaded pink). The map also shows the habitat characteristics of the study area comprising a seagrass meadow of *Posidonia oceanica* (dark blue) with patches of sand (light blue). Isobaths show site depth in meters. Yellow crosses indicate the locations where the individuals were caught and released after tagging. Note in the map the two motorboats cruising the study area. Satellite image from the Spanish public database of the National Center for Geographic Information (CNIG) edited using ArcGis v.10.6 developed by Esri (https://www.esri.com/es/arcgis).

To explore the performance of our SSM and test our hypothesis in a real case study, we used tracking data obtained from a standard acoustic tracking experiment carried out in the marine protected area of the Palma Bay, Mallorca, Spain (39**° **28′ 17ʺ N, 2**° **43′ 22ʺ E). In July 2017, an array of 14 acoustic receivers (model VEMCO VR2W, Innovasea Systems Inc., Bedford Canada) was deployed at the centre of a no-take area within a sea grass meadow dominated by *Posidonia oceanica* (L.) Delile with sand patches, the typical habitat of our study species *S. scriba*^42^(Fig. 4). The average depth of the study site was 13 m (Fig. 4). The receivers were distributed in a 150 × 150 m grid allowing for a high detection overlap among receivers, which is expected to improve the estimation of the centre of activities^26^ (Fig. 4). The Receiver’s detection range was previously measured and determined to be greater than 300 m, with a 50% probability of detection located at 150 m^38^. Each receiver was suspended 2 m above the bottom to optimize detections and avoid the thermocline effects (see details in^38^).

After the receiver array was deployed, 15 *S. scriba* were caught during one angling session, using convectional hook-and-line gear and small-sized shrimps as bait, and were transported to the Laboratory for Fisheries Experimentation and Aquaculture (LIMIA) field station in Andratx (Mallorca) for tagging. At the field station, fish were placed in a 400 L aerated fish-holding tank for recovery. After one week, individuals were anesthetized by immersion in 10 L of filtered sterile seawater with 100 mg L^−1^ of tricaine methanesulfonate (MS222, Sigma-Aldrich Inc., Missouri United States). Individuals were measured (total length in mm and weight in g) and a micro acoustic transmitter (model V7 VEMCO, Innovasea Inc., Bedford Canada; hereafter referred to as tag) was implanted into the peritoneal cavity through a dorso-ventral incision and posteriorly sutured using non-absorbable stitches (Mersilk, 4-0, Ethicon Inc., New Jersey United States). The entire surgical process was carried out within 5 min. The tags used were 20 mm in length, 7 mm in diameter, weighted 1 g in water, did not exceed a 2% of the fish’s body weight and had a manufacturer’s expected lifespan of 205 days. Every 120 s each tag emitted a specific sequence of ultrasonic pulses (acoustic signal) at a given frequency (69 kHz), which was detected by one or more acoustic receivers, allowing for individual fish detection and recognition^43,44^. After manipulation, fish were placed in a tank to recover until normal behaviour was observed and were released at the same capture location (Fig. 4). Capture, tagging and tracking of the individuals was performed following the local guidelines and regulations for animal experimentation and welfare. Experimental protocols were approved by the responsible institution for animal care (University of the Balearic Islands) and authorized by the Fisheries Department of the Government of the Balearic Islands (Experimental ethical protocol number: CEEA 60/09/16).

To simultaneously collect noise data, an underwater acoustic recorder (SNAP, Loggerhead Instruments Inc., Florida United States; sampling rate 44.1 kHz, 16 bit) provided with an omnidirectional HTI96-min hydrophone (High Tec, Inc., Mississippi United States; sensitivity − 170 dB re 1 V μPa^−1^, recording .wav files) was moored at the centre of the study area (hereafter referred to as “sound recorder”). The sound recorder was programmed to record 1 min every 11 min from June 22nd to August 8th. We used ten days of data (July 24th to August 3rd), when we obtained simultaneous data for the maximum number of tagged individuals (N = 8 individuals) and sound recordings. To automatically detect peaks of noise generated by motorboats passing at close range, we processed the data in MATLAB (version 2011b) using custom-written scripts following Merchant et al.^45^. Power spectral density (dB re 1 μPa^2^ Hz^−1^) was calculated in 1-s non-overlapping segments over the entire frequency range (i.e., 0–22 kHz) for the whole file. Following Merchant et al.^45^, we calculated the sound pressure level (SPL) as the mean squared sound pressure (p^2^_rms_) expressed in decibels, where p_ref_ is a reference pressure of 1 μPa.

$$\mathrm{SPL}=10\cdot {log}_{10}\left(\frac{{p}_{rms}^{2}}{{p}_{ref}^{2}}\right)$$

**Figure 5:**
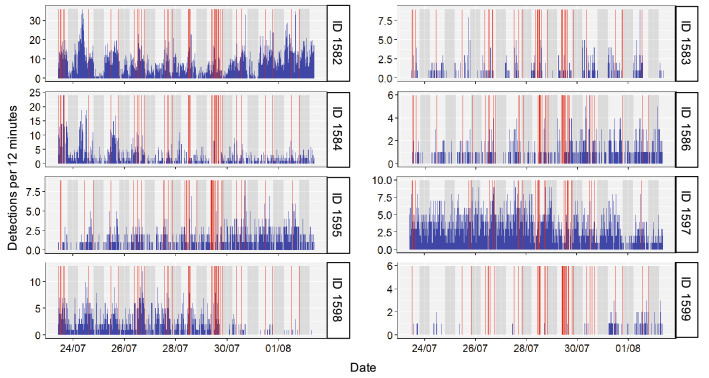
Temporal series of acoustic detections (presence of noise peaks generated by passing motorboats at close range in red and fish detections in blue) during the 10 days of the study for each fish (based on 12-min time steps). Red vertical lines represent the detection of at least a motorboat passing at close range within the 12-min time period according to our Sound Pressure Level analysis. Note the increase of motorboat detections during the weekend (July 29th and 30th). Blue bars represent number of detections for each individual by all the receivers during 12 min. Grey shadowed areas represent night time according to local sunset and sunrise data.

For the purpose of this study, we were interested in evaluating the effect of noise peaks emitted by motorboats passing at close range (within our study area). Therefore, instead of directly using SPL values, we derived a qualitative binary variable (presence vs. absence of noise peaks). To obtain such variable, we selected the audio tracks in which the SPL was at least 1.8 times higher than the basal ambient noise, defined as the constant noise recorded when boats were not present (hereafter referred to as “noise threshold”). The noise threshold was then used as a criterion to automatically detect a peak of noise result of a motorboat passing at close range. To validate the quality of the data obtained, recordings were visually inspected for detecting the presence of noise peaks generated by motorboats passing at close range (see Supplementary Information Figure S1). A total of 924 audio tracks were manually inspected, and noise peaks generated by motorboats passing at close range were detected in 79 of them. In all these 79 tracks, SPL exceeded the noise threshold, suggesting that this threshold was accurate in detecting noise peaks generated by motorboats passing at close range. The noise data obtained and used for the SSM was therefore a two-component discrete variable, presence vs. absence of noise peaks generated by motorboats passing at close range (Fig. 5).

### Estimating SSM parameters given the data

All the parameters of the integral SSM (Supplementary Information, Table S1) were estimated using a Bayesian approach. JAGS^46^ (http://mcmc-jags.sourceforge.net/) and the r2jags package^47^ were used to obtain the samples from the joint posterior distribution for the model parameters given the data (number of detections per time unit defined as 12-min time steps as imposed by the noise data collection method, day vs. night and presence vs. absence of motorboat noise). A custom R^48^ script was implemented for moving three independent MCMC chains. Technicalities of the model implementation are detailed in the custom R script, available along with the input data in the DIGITAL.CSIC repository (http://hdl.handle.net/10261/220615). An independent analysis was completed for each individual (N = 8). Convergence was assessed by visual inspection and was tested using the Gelman-Rubin statistic^49^. Values smaller than 1.1 in the Gelman-Rubin Statistic were assumed to suggest convergence^50^. Concerning priors, a truncated normal distribution between − 20 and 20 (at the logit scale) was assumed for all *p*_*n*_ and *q*_*n*_, which is virtually equivalent to a uniform-flat prior distribution between 0 and 1. For the parameters of the logistic dependence of detection probability on fish-receiver distance, the inflexion point was assumed to be uniformly distributed between − 1000 and 200 m, according with previous results using the same type of receivers^38^. This data was used as sensible initial values.

The prior distribution for the BRW parameters was set following Alós et al.^30^. The *radius* of the home range was assumed to be uniformly distributed between 50 and 1000 m, and the exploration parameter *k* (strength of the driving force attracting the individual to the centre of the home range) was assumed to be gamma distributed with parameters shape = 0.1 and scale = 0.1. Finally, the centre of the home range was assumed to be within 1000 m around the centre of the receiver’s grid, which is in accordance with the averaged receiver’s position after weighing for the number of detections. The posterior distribution of the parameters was estimated from 10,000 valid iterations after appropriate burning (the first 1,000 iterations were discarded) and thinning (one out of 10 iterations were kept). The extent of the overlap among the Bayesian Credibility Intervals (BIC) was used to test the working hypothesis, whether or not the presence of noise peaks generated by passing motorboats at close range is affecting the transition matrix.

## Results

### Acoustic tracking and noise data

All fish (N = 8) were detected every day during the 10 days considered, suggesting a high residency pattern and small space use (Fig. 5). Fish showed large among individual differences in the detection patterns. The total number of detections ranged from 10,764 to 189 detections, with an average of 3,005 detections and standard deviation of 3416 (Table 1). A natural day night regime of detections was evident for almost all fish, the number of detections notably decreased at night as previously described for this species^38^. The total number of diurnal detections was 18,006, while the total number of nocturnal detections was 6035 (Fig. 5). From the noise recording data, a total of 79 audio tracks confirmed the presence of noise peaks generated by motorboats passing at close range. The noise peaks generated by each motorboat exceeded the noise threshold to a different extent; the SPL measured exceeded the noise threshold with a minimum of 5.1 dB re 1 μPa^2^ Hz^−1^ and a maximum of 22.4 dB re 1 μPa^2^ Hz^−1^. Passing motorboats presented a temporal dynamic within the day and week (Fig. 5). At night, the number of passing motorboats detected was very limited. Furthermore, half of the motorboats detected were recorded during the weekend, which is probably linked with the high number of recreational boats present in the studied location. When combining fish tracking data with motorboat noise recordings, fish detections were discretized in 12-min periods (dictated by the noise recording data) on a temporal series that overlapped the detected motorboat activity and the natural diel pattern (Fig. 5).

**Table 1:** Fish biometrics and tracking data detections during the study period.

ID	W (g)	TL (cm)	Depth (m)	Mean detections (per 12 min)	Mean receptors (per 12 min)	Total detections
1582	–	–	–	8.97	2.75	10,764
1583	43.0	14.6	7.5	0.51	0.31	661
1584	63.1	16.4	15	2.72	1.20	3260
1586	52.9	16.0	9	0.82	0.51	988
1595	49.7	15.5	-	1.39	0.68	1667
1597	61.6	15.9	14	3.55	0.94	4259
1598	76.2	18.0	9	1.88	0.83	2253
1599	77.8	18.2	13	0.16	0.14	189

By columns: individual unique identifier (ID); biometric measures for each fish, weight in grams and total length in centimetres (W and TL respectively); capture and release depth in meters (Depth); mean number of detections every 12 min per individual (Mean detections); mean number of receptors that detected each individual every 12 min (Mean receptors); total number of detections per individual during the 10 days of the study period (Total detections). (−) Means no data available.

### State-space model

**Figure 6:**
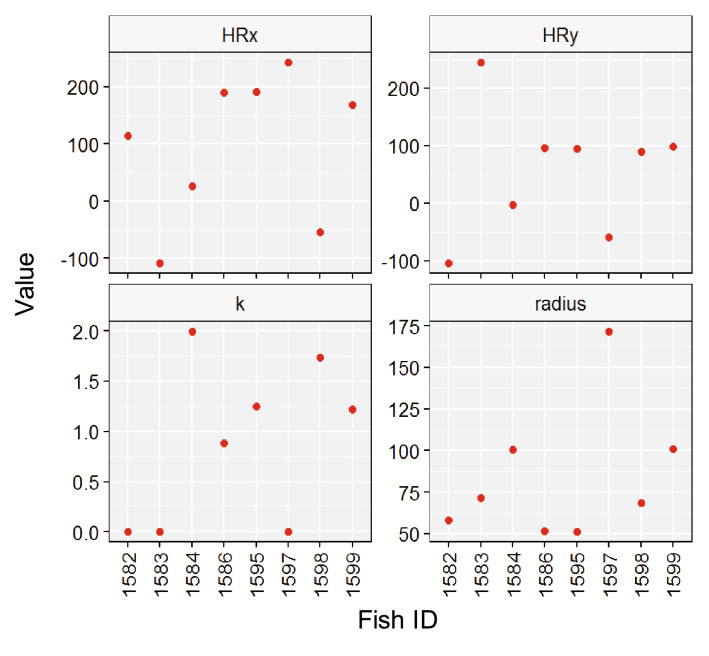
Means and Bayesian Credibility Interval (BCI) for the movement parameters estimated by the State Space Model for each individual. HRx and HRy define the centre of the home range, *k* is a measure of the driving force attracting the individual to the centre of the home range and *radius* defines the radius of the home range.

Using our SSM approach, we were able to estimate the individual movement parameters (latitude and longitude of the centre of the home range, exploration (*k*) and *radius* of the home range, Fig. 6). Regarding the latitude and longitude of the centre of the home range, fish had different centres of activity distributed across the array of acoustic receivers, although most located their centre of activity in the northern-east part (a shallower part) of the study area (Fig. 6). Curiously, fish 1586 and 1595 had practically the same position in booth coordinate axes suggesting a high overlapped space use (Fig. 6). On the general properties of the exploration of the home range, the results showed large among individual variability of *k* with a median of 0.88 s^−1^ and SD 0.80 s^−1^ with a maximum of 1.99 s^−1^ and a minimum of 5.4 10^–6^ s^−1^ (Fig. 6). The SSM revealed large differences in the attraction force to the centre of the home range with some individuals covering the whole home range in few hours while others needed days. Similar results were found for the size (*radius*) of the home range (Fig. 6). The *radius* (in m) varied among individuals with a mean of 84.25 m and SD 40.4 m, being the maximum size of the home range 175 m and the minimum 50 m. These results suggest large among individual variability in the movement of *S. scriba,* considering that all tracked fish inhabited the same area and were of similar sizes (Table1; all fish were adults).

**Figure 7:**
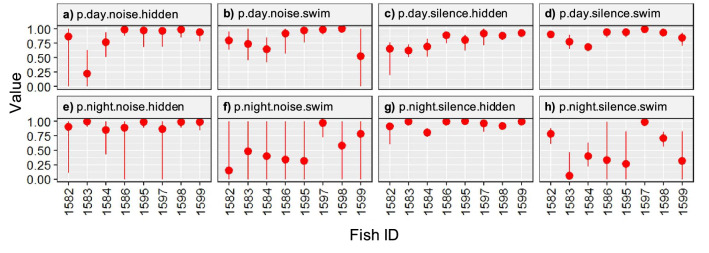
Means and Bayesian Credibility Interval (BCI) for behavioural values estimated by the Space State Model. p represents the probability of remaining in the same state (hidden or swimming). (**a**) p during the day in presence of noise peaks generated by passing motorboats when the fish is hidden. (**b**) p during the day in presence of noise peaks generated by passing motorboats when the fish is swimming. (**c**) p during the day in absence of noise peaks generated by passing motorboats when the fish is hidden. (**d**) p during the day in absence of noise peaks generated by passing motorboats when the fish is swimming. (**e**) p at night in presence of noise peaks generated by passing motorboats when a fish is hidden. (**f**) p at night in presence of noise peaks generated by passing motorboats when a fish is swimming. (**g**) p at night in absence of noise peaks generated by motorboats when the fish is hidden. (**h**) p at night in absence of noise peaks generated by motorboats when the fish is swimming.

Transitioning probabilities between behavioural states (swimming vs. hidden) in relation to the diel pattern (day vs. night) show that fish tend to remain in the same state during the day (Fig. 7a–d) and switch from swimming to hidden during the night (Fig. 7e–h). On the effects of motorboat noise, there was no clear tendency to switch between states in presence (Fig. 7a,b,e,f) or absence (Fig. 7c,d,g,h) of noise peaks generated by motorboats passing at close range, suggesting a lack of effect of this variable on fish behaviour (neither a fear effect inducing the fish to hide into a refuge, nor an attraction effect inducing the fish to leave the refuge and start swimming) under the studied conditions. When analysing the interaction between diel pattern (day vs. night) and motorboat noise (presence vs. absence of motorboats passing at close range), we found that individuals tended to remain in the same state (swimming or hidden) irrespective of the later environmental variable (Fig. 7). In general, the probability of remaining in the same state (p) is close to 1 across all individuals (Fig. 7). However, individuals that were swimming at night had a smaller probability to remain in the same state, thus tended to switch to a hidden state regardless of the presence or absence of noise peaks generated by passing motorboats (Fig. 7f,h).

## Discussion

In this work, we developed and implemented a SSM to study the effects of motorboat noise on the movement behaviour of a marine costal fish species in the wild tracked by acoustic tracking. We did not find evidence that peaks of noise generated by motorboats passing at close range are affecting the transition between swimming and hiding in *S. scriba*, although some individual peculiarities were found. Overall, the SSM modelling approach presented in this work can provide an analytical solution to study the ecological consequences of noise pollution on wild animal movement behaviour recorded via tracking. The SSM we developed here is flexible and future developments of the model can easily accommodate other types of movement behaviours fitting the specificities of the tracked species (e.g., correlated random walks following an environmental driver^51^). In addition, the inclusion of sentinel or control tags (fixed tags moored within the array that are not attached to moving fish^52^) to continuously monitor (each time step) the shape of the logistic function described by the probability of detection against disturbances, would allow to take into account the natural fluctuations of the detection probability over time. Both, a more complex movement model including different behavioural states and enhanced measurement of the positional error constitute the next steps to further develop and improve this model.

The movement pattern results obtained from applying the SSM to our field data revealed important among individual differences in the behaviour of *S. scriba*. In general, we found that our population of *S. scriba* is sedentary, making it more vulnerable at being exposed to human impacts such as motorboat noise in coastal areas^53^. We determined an average home range size of 0.02 Km^2^. This value is smaller than those reported in previous studies in *S. scriba*^38^, and other Serranidae^54^, which have reported average home range areas of ~ 1 Km^2^. However, in both cases, traditional acoustic tracking measuring individual presence vs. absence without applying a movement model was used to estimate the home range area. Our findings suggest that the SMM approach enhances the accuracy of home range size estimation via tracking. We found large among individual variability in home range sizes (ranging from 50 to 175 m). The ecological and evolutionary implications of intraspecific variability in home range size have been of great interest in the behavioural ecology research^33,55,56^. Differences in home range area could emerge from different behavioural types making some individuals more risk prone and explorative than others^40^. Furthermore, the attraction force to the centre of the home range (*k*) can be used as a proxy for exploratory behaviour. In our study species, we found high among individual variability in the time spent covering the home range ranging from few hours to several days. Overall, the exploration (*k*) extracted from our model is bigger than the one proposed in Palmer et al.^35^ for *Coris julis*, a marine Labrid species that is also highly resident. These results suggest a relatively large exploration range for our study species. Our SSM also revealed that *S. scriba* is a diurnal species, being more active during the day than at night (Fig. 5), a usual characteristic of species inhabiting tropical and temperate areas^57,58^. Therefore, our work provides additional support to the results found by March et al.^38^ on the diel pattern of behaviour of *S. scriba*.

Regarding the results of noise peaks generated by passing motorboats (Fig. 5), we found the expected dynamic across time with more motorboat detections during the day than at night. Among-day variation showed an increase in the number of motorboats during the weekend, likely related to recreational activities in the studied coastal area. There was no evident effect of noise peaks produced by motorboats passing at close range on the *S. scriba* movement pattern of behaviour. However, the lack of effect of noise peaks generated by motorboats on fish behaviour needs to be contextualized under the specific studied circumstances.

First, we only considered the effects of noise peaks generated by motorboats passing at close range, we did not measure overall effects of noise pollution. Furthermore, the behaviour studied in this work is limited to the differential transition between states, swimming and hidden. Including other behaviours such as swimming speed and position of the fish in the water column could provide more information on the effects of noise pollution on movement behaviour. For these reasons, we cannot exclude generalized effects of motorboat noise on behaviour, physiology and overall fitness of the studied species. Next steps to improve our model should include other behaviours to broaden the scope of the effects of noise pollution on fish behaviour.

Second, the relatively large number of motorboats passing through the study area might have caused a habituation effect in our study species. This habituation phenomenon has already been observed in other species; fish in habitats with higher motorboat disturbance show reduced sensitivity to motorboat noise^59^. Furthermore, Holmes et al.^60^ found that some coastal fishes returned to pre-disruption behaviour after 20 min of motorboat noise exposure. However, behavioural habituation to continuous sounds of heavy ship traffic may hide physiological stress with a potential impact on survival and reproduction^60–63^, and general population maintenance^15^. To demonstrate the validity of the habituation hypothesis in our species, fish could be subject of experimental trials in controlled environments (aquaria or tanks), where individuals from high and low acoustically polluted areas are exposed to different levels of noise. In this way, sound tolerance and habituation could be measured in *S. scriba* when other environmental factors are controlled (habitat, population density and oceanography).

Third, our methodological approach had some limitations that could hide a real effect of motorboat noise on behaviour. It is possible that we failed to detect changes in behaviour due to the relatively sparse temporal resolution of tracking and discrete noise recordings (12-min time steps). For example, a motorboat could have passed just before the sound recorder started the one-minute recording period, affecting the general behaviour of the fish but not being considered in the noise data. An increase in resolution of the tracking data (e.g. larger sample size during a longer period of time and more accurate positioning) and a more detailed noise dataset (e.g. continuous sound recording of noise levels in dB) could provide a more realistic view into the behaviour of the studied species. To amend this limitation, we provided our SSM with an extension that accommodates to fit a continuous explanatory variable (e.g. noise levels in dB). Because of the nature of our empirical data, we were not able to use this addition to the model, however this can be used in the future to obtain stronger analytical results on the effects of noise on fish behaviour.

Fourth, effects of motorboat noise on behavioural responses can be different depending on the engine types and motorboat speed^64^. Furthermore, different sounds can trigger different behavioural responses on animals^6^. Currently, there are no available studies describing the hearing range and threshold of *S. scriba,* which is likely sensitive only to the particle motion component of anthropogenic noise. Implementing a set of experiments under laboratory conditions with fish exposed to different sounds and different levels of noise pollution could help determine the best noise type and sound frequency interval affecting the behaviour of *S. scriba*, thus allowing us to target specific sounds when analysing natural recordings in following wild-based experiments.

The noise generated by commercial and recreational motorboats is probably the most important source of anthropogenic noise in coastal areas^5,6^. Studies on the effects of noise pollution on fish behaviour are important, especially for marine protected areas management. Literature showing the negative effects of noise pollution over all marine fauna, mammals, fishes and invertebrates is abundant^8,23,65,66^, and key for understanding the effects of such pollution on the marine environment as a whole^12^. We were not able to find an effect of noise peaks generated by motorboats passing at close range on a specific behaviour of *S. scriba*, but this does not imply that the activity of motorboats is not affecting the functioning of the ecosystem enclosed in the Palma Bay marine protected area. Overall, this work provides important analytical solutions for the study of the ecological impacts of anthropogenic noise pollution in wild fish populations. Thus, delivering a tool for future studies and managerial applications.

## Supplementary Information

Supplementary Information

## Data Availability

The datasets generated and analysed during the current study are available at the DIGITAL.CISC online repository (http://hdl.handle.net/10261/220615), other information is included in this article as supplementary information.
