# Supplementary material for: A state-space model to derive motorboat noise effects on fish movement from acoustic tracking data
Source: Sci Rep. 2021 Feb 26;11:4765. doi: 10.1038/s41598-021-84261-2 (PMC7910575; doi:10.1038/s41598-021-84261-2)
Supplement: Supplementary file 1 — Supplementary Information [file 41598_2021_84261_MOESM1_ESM.pdf]

A State-Space model to derive motorboat noise effects on fish movement from acoustic tracking data

Supplementary Information

Margarida Barcelo-Serra, Sebastià Cabanellas, Miquel Palmer, Marta Bolgan, Josep Alós

Supplementary Table S1. Parameters and variables of the State-Space Model (SSM).

Parameters and variables	Description
HRx	Location of the centre of the home range (X coordinates)
HRy	Location of the centre of the home range (Y coordinates)
b[1]	Slope of the detection curve when the fish is swimming
b[2]	Slope of the detection curve when the fish is hidden
inf[1]	Distance of inflection (50% detections) of the detection curve when the fish is swimming
inf[2]	Distance of inflection (50% detections) of the detection curve when the fish is hidden
k	Attraction force to the centre of the home range ($seconds^{-1}$)
p.day.noise.hidden	Probability of continuing hidden during the day when a motorboat is passing at close range
p.day.noise.swimming	Probability of continuing swimming during the day when a motorboat is passing at close range
p.day.silence.hidden	Probability of continuing hidden during the day in absence of passing motorboats
p.day.silence.swimming	Probability of continuing swimming during the day in absence of passing motorboats
p.nigth.noise.hidden	Probability of continuing hidden during the night when a motorboat is passing at close range
p.nigth.noise.swimming	Probability of continuing swimming during the night when a motorboat is passing at close range
p.nigth.silence.hidden	Probability of continuing hidden during the night in absence of passing motorboats
p.nigth.silence.swimming	Probability of continuing swimming during the night in absence of passing motorboats
radius	Radius of a circular home range (meters).
sd.stop	Short movements of the fish when is hidden

a)

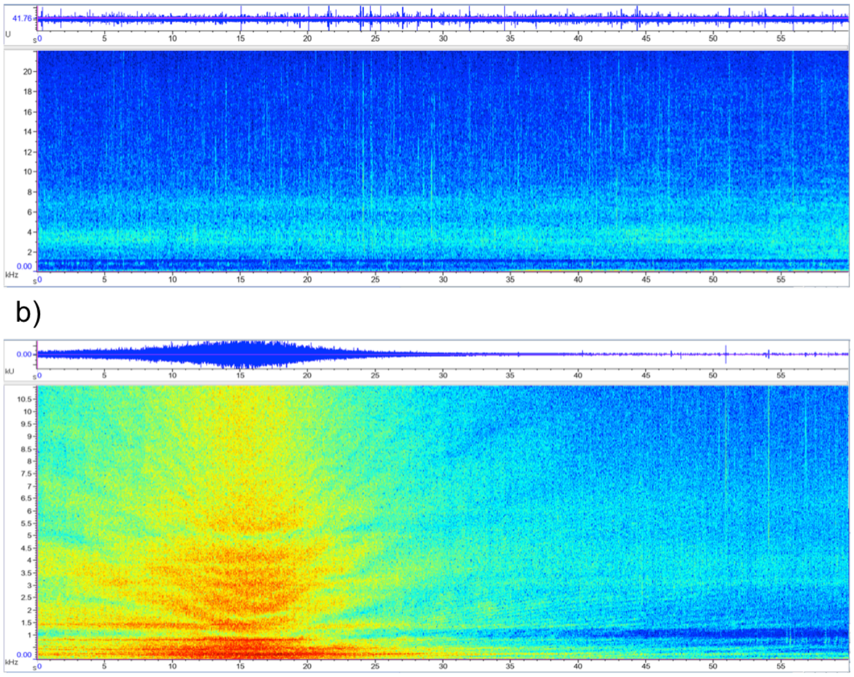

Supplementary Figure S1 Waveform and spectrogram (Hanning window, 512 FFT, 50% overlap) of two audio files. a) Background noise occurring during the day in presence of boat noise but without motorboats passing at close range and b) Peak of noise emitted by a motorboat passing at close range.
